# Decompressive craniectomy: indications and results of 24 cases at the neurosurgery clinic of Fann university hospital of Dakar

**DOI:** 10.11604/pamj.2021.38.399.27571

**Published:** 2021-04-26

**Authors:** El Hadji Cheikh Ndiaye Sy, Yakhya Cisse, Alioune Badara Thiam, Louncény Fatoumata Barry, Maguette Mbaye, Abdoulaye Diop, Mbaye Thioub, Mohameth Faye, Attoumane Fahad, Mouhamadou Moustapha Ndongo, Ansaou Aboudou Soilihi, Nantenin Doumbia, Momar Codé Ba, Seydou Boubacar Badiane

**Affiliations:** 1 Neurosurgery Department, Fann University Hospital Center, Dakar, Senegal,; 2 Neurosurgery Unit, Ziguinchor Regional Hospital, Ziguinchor, Senegal,; 3 Neurosurgery Department, Idrissa Pouye General Hospital, Dakar, Senegal

**Keywords:** Craniectomy, decompression, infarction, trauma

## Abstract

Decompressive craniectomy is a surgical technique considered to be the last step in the management of intracranial hypertension. The objective of our study was to evaluate our results in the management of intracranial hypertension by decompressive craniectomy. This was a retrospective study of 24 cases of decompressive craniectomy performed over a 9-year period (from January 2010 to December 2019) at the Fann Neurosurgery Clinic. The mean age of the patients was 33.82 years, there was a male predominance with a sex ratio of 2.42. The most frequent indication was severe cranioencephalic trauma with 50%. The cerebral computed tomography (CT) scan was the key examination and was performed in all our patients. Complications were entirely infectious and were the cause of 73.33% of deaths. Thirty-five percent of the patients had received prior treatment before the decompressive craniectomy. The functional prognosis was good in 44.44% of cases, moderate in 33.33% of cases, 1 (11.11%) patient had a severe disability and 1 (11.11%) patient was in a vegetative state. Mortality rate was 62.5% of patients in our study series. Despite the lack of sophisticated techniques for diagnosis and monitoring of intracranial hypertension, our results remain acceptable with 37.5% survival. The early completion of this surgery allows us to be more efficient with a significant reduction in morbidity and mortality.

## Introduction

Practiced since the beginning of the 20^th^ century [1], decompressive craniectomy (DC) is a technique proposed in the treatment of intracranial hypertension (ICH) secondary to cerebral attacks. Its use appears to be associated with an improvement in the vital and functional prognosis of survivors [2]. Nevertheless, it seems to be accepted in a very heterogeneous way despite the accumulation of scientific data and international recommendations [3, 4]. It is performed in victims of traumatic brain injury, stroke and other conditions associated with high intracranial pressure (ICP) as in some tumor cases [5]. It may also be indicated in rare cases in the management of craniostenosis and infectious diseases (subdural empyema, encephalitis and toxoplasmosis) [6-8]. The objective of our study was to evaluate our results in the management of ICH by decompressive craniectomy.

## Methods

We retrospectively studied over 9 years (from January 2010 to December 2019) the records of 24 patients who had undergone decompressive craniectomy at the neurosurgery clinic of the Fann CHUN in Dakar, Senegal. The parameters studied were frequency, age, sex, history, initial Glasgow score, initial Blantyre score, pupillary signs of involvement, initial neurological signs, CT scan results, etiologies, indications, time to management, surgical technique, duration of surgery, complications, mortality, Glasgow Outcome Scale (GOS). The data collected was analyzed using SPSS version 21.0 software.

## Results

**Epidemiological characteristics:** the frequency was 2.44 cases per year. The mean age was 33.82 years with extreme ages ranging from 40 days to 60 years. The sex ratio was 2.42 (17H/7F). Three of the patients were known hypertensive, poorly monitored, two were known epileptic, poorly monitored, one patient had a history of marsupialization and craniopharyngioma removal. The other patients had no particular terrain or history.

**Clinical manifestations:** the initial Glasgow score (GCS) was assessed in 22 patients and the initial Blantyre score in 2 infants. Table 1 summarizes the initial Glasgow and Blantyre scores. Eleven (11) patients had a pupillary abnormality: 7 cases of anisocoria and 4 cases of reactive bilateral mydriasis. The associated neurological signs were: convulsive seizures in 6 cases, generalized in 5 cases and focal in 1 case, six (6) patients presented a pyramidal syndrome of hemiplegic type.

**Table 1:** distribution of cases by GCS and/or Blantyre score

Number	Glasgow score
8	≤07
14	8-11
**Number**	**Score of Blantyre**
2	1/5

**Figure 1:**
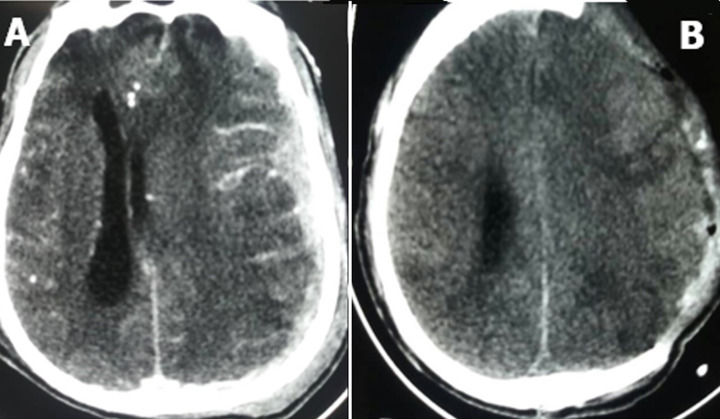
A) axial section brain CT scan showing acute subdural hematoma; B) post-operative cerebral CT scan of a decompressive craniectomy for an acute subdural hematoma in the same patient

**Figure 2:**
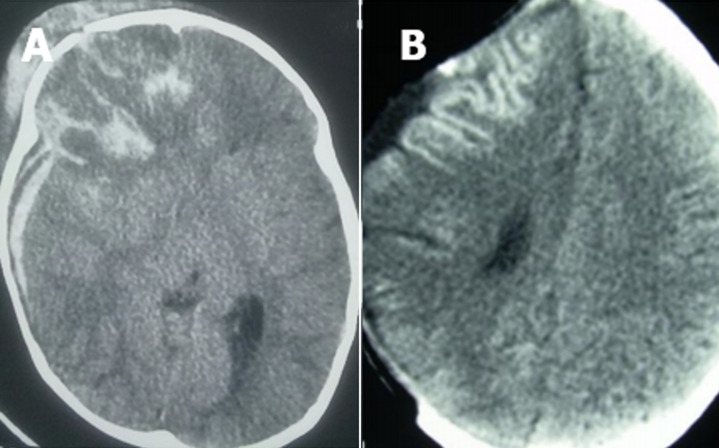
A) pre-operative axial section brain CT scan of a right oedemato-hemorrhagic frontal contusion associated with temporal (HSDA); B) decompressive post-craniectomy brain CT scan of right oedemato-hemorrhagic frontal contusion associated with temporal HSDA in the same patient

**Figure 3:**
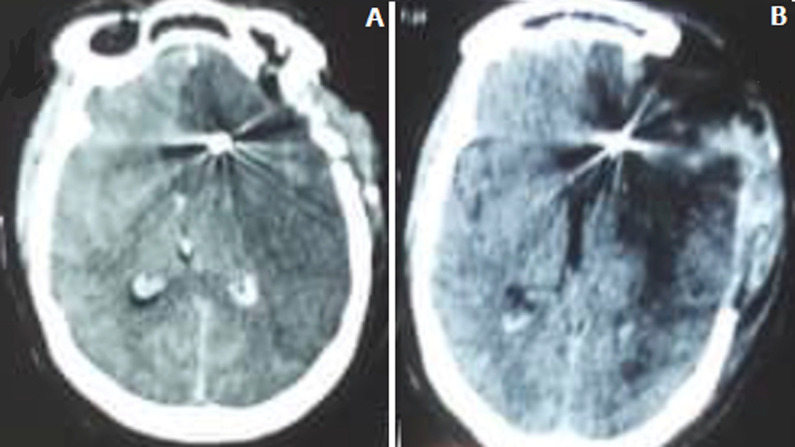
A) cerebral CT scan in preoperative axial section of a massive left sylvian infarction after clipping a carotid aneurysm; B) decompressive post-craniectomy brain CT scan of left massive sylvian infarction after aneurysm clipping in the same patient

**Figure 4:**
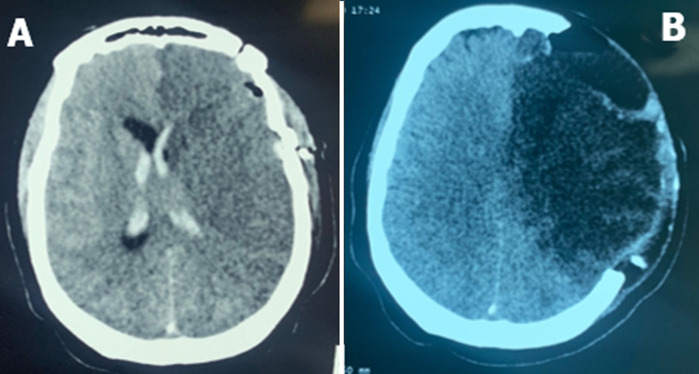
A) axial section of a brain CT scan of a post-exercise ischemia of an olfactory meningioma; B) cerebral CT scan axial section post-craniectomy decompressive ischemia post-exeresis of olfactory meningioma

**Figure 5:**
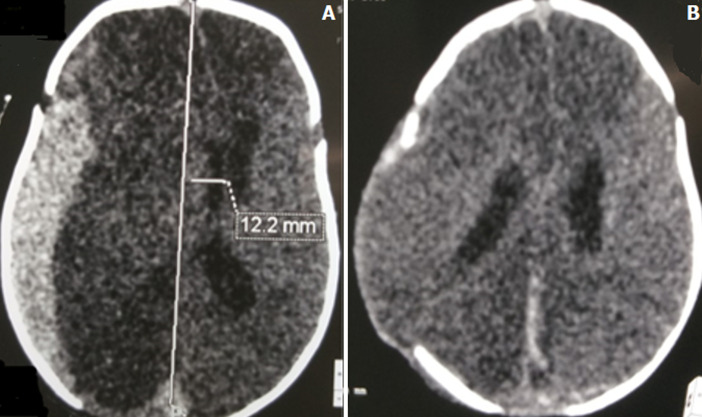
A) axial section brain CT scan of an HSDA and post ventricular puncture ischemia; B) cerebral CT scan axial section after decompressive HSDA and post-ventricular puncture ischemia

**Figure 6:**
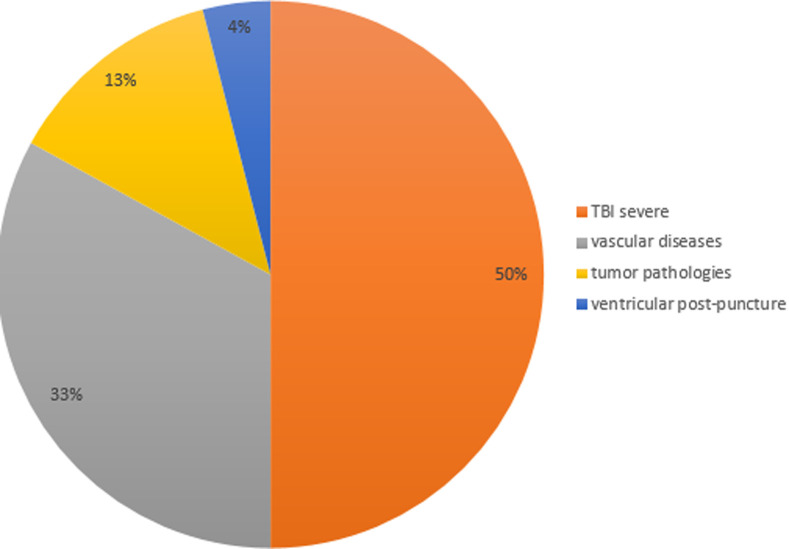
etiologies according to their percentage

**Medical imaging data:** brain computed tomography (CT) scan was the main complementary examination performed on patients in our series. In the 12 cases of traumatic brain injury (TBI) it showed: 7 cases of acute subdural hematoma (ASDH) (Figure 1), 3 cases of cerebral contusions (Figure 2) and 2 cases of diffuse cerebral edema. In the 8 cases of vascular pathologies, it showed: 2 cases of post-clippage ischemia of the sylvian artery (Figure 3), 2 cases of malignant sylvian infarction in epilepsy, 3 cases of malignant sylvian infarction in hypertension and 1 case of cerebral oedema after resection of an arteriovenous malformation (AVM). Regarding tumor pathology, she found 2 cases of sylvian artery ischemia after resection of a craniopharyngioma and an olfactory meningioma (Figure 4) and 1 case of cerebral edema after resection of a scythe meningioma of the brain. CT also showed acute subdural hematoma (ASDH), associated with hemispheric ischemia complicating ventricular puncture in an infant (Figure 5). The etiologies are summarized in Figure 6.

***Indications:*** the average time of care was 3.2 days with a minimum of 24 hours and a maximum of 16 days. In sixty-five percent (65%) of cases, CEP was immediate. Patients were operated on the same day they were hospitalized. Thirty-five percent (35%), however, had prior treatment (1 case of external ventricular shunt and 6 cases of medical treatment). The indications are summarized in Table 2.

**Table 2:** indications for decompressive craniectomy

Type of lesions	Number	Percentage %
Malignant Cerebral Infarction	8	33.33
ASDH	8	33.33
ASDH + malignant ischemia	1	4.16
Diffuse cerebral edema	4	16.66
Brain contusions	3	12.5

**Surgical techniques:** the decompressive flaps performed in our patients were fronto-temporo-parietal (curvilinear hemicraniectomy) in relation to the lesions. In 60% of cases, cranioplasty was performed about 4 weeks after surgery. The average duration of the surgery was 2h 30, with a minimum of 1h 30 and a maximum of 5h 30. The average length of hospitalization was 20 days, with a minimum of 1 day and a maximum of 80 days.

**Evolution:** in our series of studies, the rate of post-craniectomy complications was not negligible. These were mainly infectious complications, including 2 cases of empyema, 1 case of ventriculitis, 6 cases of pneumopathies and 4 cases of surgical site infection. Mortality was 62.5% or 15 deaths with (8 cases of traumatic origin, 3 cases of tumor origin, 3 cases of vascular origin and 1 case of post-ventricular puncture origin). Of the 15 deaths, 11 (73.33%) were due to a complication after craniectomy and 4 (26.66%) deaths were of unknown cause. Survival was 37.5% or 9 cases with Glasgow Out Scale (GOS) to 5 in 4 (44.44%) patients, to 4 in 3 (33.33%) patients, to 3 in 1 (11.11%) patient and to 2 in 1 (11.11%) patient.

## Discussion

Decompressive craniectomy is a radical surgery in the treatment of ICH, allowing the expansion of brain volume and resulting in a decrease in intracranial pressure [9]. Out of 24 cases of decompressive craniectomy performed over a period of 9 years, i.e. 2.44 cases per year with a peak in 2018 (6 cases), our study involved subjects between 40 days and 60 years of age with an average age of 33.82 years and a sex ratio of 2.42. Several studies have been conducted over the last twenty years. They were often retrospective studies, involving small series of patients. The results were highly variable due to a very heterogeneous population, of varying ages, including as many women as men [10-14]. We note that our series joins the different series published in the literature concerning the heterogeneity of the population.

Clinically, the initial Glasgow and/or Blantyre score, as well as the presence of a sign of commitment would influence the vital prognosis. In our study, in the 8 patients (33.33%) with SCT ≤ 7 at the time of management we noted 6 deaths or 75%, 7 deaths or 50% in the 14 patients (65%) with SCT > 7 and we noted 2 deaths in infants with Blantyre a 1/5. Ucar *et al*. [15] made a comparison between two groups of patients treated by decompressive craniectomy but only in the context of severe trauma. Group I consisted of 60 patients with a SCT between 4 and 5 and group II consisted of 40 patients with a SCT between 6 and 8. They found unfavorable results in 96.6% of group I patients versus 65% of group II patients, and favorable results in 3.4% in group I and 25% in group II. This would explain the influence of the initial SCT on the vital prognosis.

To establish the diagnosis of ICH, ventriculostomy with intraventricular catheter placement certainly seems to be the most efficient method, since it allows at the same time the cerebrospinal fluid (CSF) to be drained. Intracranial pressure (ICP) can also be measured using an intra parenchymal sensor [16]. All these types of monitoring do not yet exist in our country. In the present study, we made the diagnosis of ICH on the basis of cerebral edema with cerebral involvement associated with a vigilance disorder with a Glasgow score < 12. Brain CT scan is the key complementary examination before any craniectomy. It has been performed on all our patients. Cerebral magnetic resonance imaging (MRI) also plays a key role in the etiological and topographical diagnosis of lesions. However, there are constraints related to the availability of the examination, its exorbitant cost in our regions, and the fact that it must be performed in an emergency before performing a decompressive craniectomy.

In our series, the indication for craniectomy was retained in 70.83% of cases after a single brain scan (in 17 of our patients). Five (20.83%) patients had benefited from two brain scans before the craniectomy and only 2 (8.33%) patients had to benefit from three brain scans. However, no MRI scan had been performed. In the case of emergency management, the brain CT scan was sufficient in this series. This shows that it is therefore possible to rely on CT scan alone for the indication of a decompressive craniectomy because the degree and severity of brain compression is well visualized.

Surgically, several studies in the literature have found favorable results regarding the usefulness of decompressive craniectomy in many neurosurgical pathologies. Historically, it has been used to treat ICH secondary to Reye's syndrome in children [2]. Stefini *et al*. reported good results after craniectomy for the treatment of hemorrhagic infarctions secondary to venous sinus thrombosis [17]. In the surgical treatment of spontaneous haematomas, Dierssen *et al*. reported a significant improvement in the mortality rate in patients treated by haematoma evacuation with craniectomy compared to a series of patients treated only by haematoma evacuation [18]. Also, many surgeons advocate the use of decompressive craniectomy in certain cases of empyema [19, 20].

A study of a series of 170 patients operated on by craniectomy showed that prognostic factors such as the urgency of the surgery and the duration of the surgery ≥ 200 minutes (3h 20min) influenced the functional and vital prognosis of the different patients [21]. In our study, 65% of the cases who received immediate CEP (the first 24 hours) had a mortality of 58.82% and 35% who received prior medical treatment had a mortality of 71.4%. This implies the short time to management as a predictor of good outcomes. The average duration of surgery was 2.5 hours, with a minimum of 1.5 hours and a maximum of 5.5 hours. Mortality of 53.3% in patients who underwent the surgery of less than 3 hours and 80% mortality in those who underwent the surgery with a duration of ≥ 3 hours. There would then be a correlation between early surgery, the duration of the intervention and the vital prognosis of the patients.

Complications of craniectomy can be divided into early complications and secondary complications. Early, bleeding complications are the main ones. Decompression and decreased ICP are likely to promote the development of a subdural or contralateral extradural hematoma [22]. In our series, none of these complications were found. Secondary complications are essentially cerebral hemodynamic. After decompressive surgery, the brain undergoes several changes concerning hemodynamics with venous drainage, CSF movement and general metabolism. This is most evident clinically in patients with improvement followed by deterioration of neurological status within a few weeks or months after craniectomy accompanied by concave depression over the flap site. This phenomenon has been described as the sinking flap syndrom. Neurological signs may be limited to fatigability, discomfort, depression, vibration intolerance, headaches and more rarely sensory and/or motor deficits.

In 1977, Yamaura and Makino [23] published a study of 33 patients with scalp flap syndrom after craniectomy, and reported neurological improvement after bone flap reimplantation in 88% of patients with moderate symptomatology. Some authors explained the pathophysiology of this phenomenon by the transmission of atmospheric pressure to the cranial cavity. Recently several authors have reported a negative gradient between atmospheric pressure and ICP. Also, drainage of the CSF in case of hydrocephalus or meningitis after craniectomy may create this pressure gradient. In addition, hemodynamic change due to prolonged dehydration may contribute to this phenomenon. Treatment of this syndrome consists of cranioplasty to reconstruct the skull [24, 25]. In their study of 23 patients undergoing early cranioplasty, within 5 to 8 weeks after craniectomy. Liang *et al*. [26] reported recovery of neurological signs in the majority of patients after cranioplasty. However, other complications are not negligible, such as subdural hygroma, hydrocephalus or cranioplasty infection [27, 28].

In our study, all post-operative complications were essentially of infectious origin and were found in 54.16% of cases. These were empyema (2 cases), ventriculitis (1 case), pneumopathy (6 cases) and surgical site infections (4 cases). Post-operative sequelae were marked by motor deficits (4 cases) and motor aphasia (1 case). In terms of prognosis, most studies, particularly recent ones, conclude that there has been a sometimes-spectacular improvement in the vital prognosis. For severe ECTs, a good response is noted after decompressive craniectomy [29]. In our study, only 33.33% of severe ECTs had a good vital prognosis. However, 73.33% of deaths were due to post-operative complications. With regard to sylvian ischemic stroke, it seems logical that craniectomy influences the mortality of patients for whom ICH represents the main risk while primary involvement is moderate. Several studies report higher mortality with conservative treatment versus increased survival after decompressive craniectomy [30-32].

In our study, of the 2 cases of post-exercise sylvian ischemia, 2 cases of post-exercise sylvian ischemia of olfactory meningioma and 1 case of post-exercise ischemia of craniopharyngioma, there was 100% mortality for post-exercise ischemic stroke and 100% survival for post-exercise ischemia. Spontaneous malignant stroke mortality was 66.6%. This would imply the etiology of the infarction as a factor conditioning mortality. We found 100% mortality in patients over 45 years of age, 26.66% in patients between 30 and 45 years of age, 40% in patients under 30 years of age, and 100% mortality in both infants. Indeed, Gupta *et al*. published a study of 138 patients treated by hemicraniectomy for massive infarction of the middle cerebral artery territory among 75 patients over 50 years old, 80% died or were severely disabled compared to only 32% of the 63 patients ≤ 50 years old [33]. While decompressive craniectomy is likely to improve the vital prognosis of severe TCE and malignant sylvian infarction, it is essential that the functional prognosis and quality of life of surviving patients be assessed.

There are several scores to judge long-term functional outcomes: Glasgow Outcome Scale (GOS) and modified Rankin Scale (mRS) are the most widely used because of their simplicity. The GOS was first developed in 1975 to evaluate functional results after decompressive craniectomy [34]. Patients are classified into: favorable prognosis (GOS 5 and 4) and unfavorable prognosis (GOS 3 and 2). The modified Rankin Scale (mRS) is mainly used in cerebral infarction [31]. The prognosis is favourable from 0 to 3 and unfavourable from 4 to 6. Numerous retrospective studies and a few randomized prospective studies conclude that the functional prognosis improves after decompressive craniectomy [32, 35].

In our series, among the 9 patients who survived, 4 (44.44%) patients had a good functional prognosis without sequelae (GOS5), (3 TCE, 1 malignant stroke), 3 (33, 33%) patients had moderate disability (GOS4) (1 severe TCE and 1 post-clippage DALY), 1 (11.11%) patient had severe disability (GOS3) (1 post-clippage DALY) and 1 (11.11%) patient was in a vegetative state (GOS2) (1 severe TCE).

**Limitations of our study:** the study was retrospective. The workforce did not allow a multivariate analysis. The population studied was very heterogeneous.

## Conclusion

Decompressive craniectomy is a surgical technique used to treat intracranial hypertension. Its effectiveness has been scientifically proven in malignant ischemic stroke. In traumatology, it is recommended in cases of intracranial hypertension refractory to medical treatment. Despite the lack of sophisticated techniques for diagnosis and monitoring of intracranial hypertension, our results remain acceptable with 40% survival. The early realization of this surgery allows us to be more efficient with a significant reduction in morbidity and mortality. The whole problem lies in the neurological evolution of craniectomized patients. Our work should be continued by a study on the quality of life of these patients, who sometimes remain severely disabled.

### What is known about this topic

- Decompressive craniectomy is the last step in the treatment of intracranial hypertension;
- Its achievement is associated with an improvement in the vital and functional prognosis of the survivors;
- It is performed in patients with brain injuries of traumatic, vascular or tumor origin complicated by intracranial hypertension.

### What this study adds

- This is the first work on decompressive craniectomy in our context;
- Our work provides baseline data on decompressive craniectomy in our context, allowing us to compare our future results with current results and will allow other authors to eventually compare their results with ours;
- Our work reports in the literature the experience and results of a country with limited health resources in the management of intracranial hypertension by decompressive craniectomy.
